# Oncology Patient Interest in the Use of New Technologies to Manage Their Disease: Cross-Sectional Survey

**DOI:** 10.2196/11006

**Published:** 2018-10-23

**Authors:** Roberto Collado-Borrell, Vicente Escudero-Vilaplana, Antonio Calles, Estela Garcia-Martin, Belen Marzal-Alfaro, Eva Gonzalez-Haba, Ana Herranz-Alonso, Maria Sanjurjo-Saez

**Affiliations:** 1 Hospital General Universitario Gregorio Marañón Madrid Spain

**Keywords:** eHealth, app, information and communication technologies, oncology patient

## Abstract

**Background:**

Information and communication technologies (ICTs) in oncology can revolutionize the medical care of cancer patients. ICTs can promote patients’ empowerment and real-time disease monitoring. There is limited information about the impact of ICTs in cancer patients or their level of interest in using these tools for greater management of their condition.

**Objective:**

This study aimed to understand the ICT usage profile in hematology-oncology patients to identify their needs and determine their level of interest in these technologies as a means of managing their disease.

**Methods:**

A 28-item questionnaire was drawn up by a multidisciplinary team including pharmacists and oncologists. The questions were organized into 3 blocks, which were as follows: block A—sociodemographic characteristics; block B—use of ICTs when searching for health-related information; and block C—usage preferences for health apps. Hematology-oncology patients receiving treatment between May and July 2017 were included. A paper copy of the questionnaire was handed over to patients in either the day hospital or the pharmaceutical care consultancy in pharmacy services.

**Results:**

A total of 650 questionnaires were handed out, with a participation of 94.0% (611/650). Patient sociodemographic characteristics were as follows*:* mean age was 57.8 years (age range: 19-91). Of 611 participants, 40.7% (249/611) had a university education, and 45.1% (276/611) of participants reported their overall state of health to be good. Results from use of ICTs when searching for health-related information were as follows: 87.1% (532/611) of participants were interested in being informed about health-related matters. Of all participants, 75.5% (532/611) sought information from health professionals and 61.3% (375/611) on the internet. Before going to their doctor’s appointment, 21.8% (133/611) of patients looked up information about their disease or treatment on the internet. This access to the internet rose to 50.9% (311/611) after their first medical appointment with their oncologist. Usage preferences for health apps were as follows: 82.7% (505/611) had a smartphone, whereas 20.3% (124/611) had a health app installed. Overall, 81.5% (498/611) would use an app if their health professional recommended it to them, but 39.6% (242/611) were not willing to pay for it.

**Conclusions:**

The hematology-oncology patients showed a great deal of interest in searching for health-related information by means of ICTs, especially using smartphones and apps. The issues that drew the most interest in terms of apps were appointment management, advice on disease management, and communication with health professionals. Free access to these features and the recommendation by a health professional are important factors when it comes to their use. Therefore, the health care provider is a key element in the recommendation of ICTs, providing their knowledge and experience concerning their correct usage.

## Introduction

Population aging, improvements in public health care, and therapeutic advances have given rise to an epidemiological pattern that is mainly represented by chronic diseases. This new patient profile introduces additional requirements in terms of patient care (as it corresponds to more detailed health information) and greater opportunities for communication and even novel telecare services. In this regard, information and communication technologies (ICTs) are bringing about a revolution in the management of chronic patients, acting as a source of information on one hand, while providing real-time monitoring for health professionals and two-way communication between both parties on the other [1-3]. The report *Los Ciudadanos ante la e-Sanidad* (Public Opinion on eHealth Care) by the Spanish National Observatory of Telecommunications and Information Society (ONTSI) estimated that 70.4% of the Spanish population owns a smartphone, and 74.4% of internet users refer to the internet for health-related information [4].

The introduction of mobile health has meant the classic features of ICTs (content, chats, and contacts) could be focused toward providing services for patients. The use of wireless and portable devices (smartphones, tablets, wearables, and smartwatches) facilitates the collection, processing, and transmission of health information of patient through mobile phone apps and remote monitoring devices. All these features can present several advantages such as developing the active role of patients in their own health care and affording health professionals’ real-time access to patient clinical information [5-8].

Oncology is one of the medical fields that could benefit the most from these new tools [3,6]. Although cancer is associated with a high rate of morbidity and mortality, ongoing advances are producing higher survival rates, converting some of these diseases from fatal to chronic conditions [9]. In addition, patients are increasingly resorting to apps in search of information given the complexity of treatment and the numerous unknowns they must confront [3,10,11].

Currently, a wide range of health apps are available, particularly ones designed especially for oncology patients [3,12]. These apps have several features and functions, from the simplest intended for accessing information, to the more complex apps that can help guide the diagnosis or perform patient follow-up [3]. However, many of the apps are developed without taking into account preferences and/or needs of a patient [10]. In fact, only 40% of those available are considered to have any value for addressing health issues, and just 2% provide two-way communication between the patient and health professional [12]. Many of these apps also suffer from a lack of scientific validity and reliability. A review conducted in November 2014 found that only 48.8% of apps designed for cancer patients had been developed by health professionals [3]. As these tools are not being regulated by the relevant institutions, the main onus falls on health professionals to ensure the validity of the apps they recommend or prescribe to their patients [2,3,10]. The first step when prescribing a quality ICT is awareness of the true impact these technologies are having on lives of patients.

Therefore, our aim was to understand the usage profile of information and communication technologies in oncology patients to identify their needs and determine their degree of interest in these technologies as a means of managing their disease.

## Methods

A 28-item questionnaire was designed by a multidisciplinary team, including pharmacists and oncologists, with experience of ICTs applied to the field of oncology. The questions were organized into the following 3 blocks: block A—sociodemographic characteristics of a patient (questions 1-9), block B—use of ICTs when searching for health-related information (questions 10-21), and block C—usage preferences for health apps (questions 22-28). The design of the questionnaire considered the model form created by ONTSI [4] and incorporated advice from a management consultancy firm specializing in process design, implementation, and improvement. With respect to the types of questions, the questionnaire comprised dichotomous (questions 2, 3, 6, 12, 18, 20, 22, and 27) with true or false response options or polytomous (questions 4-5, 7-10, 11, 13-17, 19, 21, and 23-26) categorical variables. A total of 10 patients completed a draft paper questionnaire to validate the fact that it was well understood.

Hematology-oncology patients in antineoplastic therapy (chemotherapy, immunotherapy, oral targeted therapies, and hormone therapy) and/or support therapy were included. Patients younger than 18 years and those who would have had trouble understanding the questions because of language or cultural barriers were excluded. Participation was voluntary and anonymous. The inclusion period ran from May to July 2017. A paper copy of the questionnaire was handed over to every patient in the day hospital (administration or waiting rooms) or the pharmaceutical care consultancy in the outpatient pharmacy services area. Alternatively, patients could take the questionnaire home and return it filled out at their next appointment.

The questionnaire was approved by the clinical research ethics committee of the hospital, project code EHPHDO2017, and conducted in accordance with the ethical principles laid down by the Declaration of Helsinki. It did not include any information about the personal data of patients to ensure data confidentiality.

Data were analyzed using the software SPSS version 21.0 (IBM Corp) for Windows. The variables were analyzed using descriptive statistics. Normality was analyzed by means of the Kolmogorov-Smirnov test. Numeric variables were compared with Student *t* test or the Mann-Whitney test. The association between qualitative variables was studied using Pearson chi-square test or Fisher exact test. Missing data were handled with simple imputation method. The corresponding measures of association and risk were calculated along with their CIs. Results with a value of *P*<.05 were considered statistically significant.

## Results

### Patients’ Sociodemographic Characteristics

A total of 650 questionnaires were handed out, with a participation of 94% (we finally analyzed 611 questionnaires). Sociodemographic characteristics of patients are shown in Table 1. The mean age of patients was 57.8 years (range: 19-91). Of all participants, 61.9% (378/611) were women, 89.5% (547/611) lived with other people, and 40.7% (249/611) had a university education. Overall, 29.2% (179/611) of cases corresponded to hematologic tumors. Among nonhematologic tumors, the most frequent were breast (124/611, 20.3%), colorectal (113/611, 18.5%), and lung (60/611, 9.8%). Over half of the patients considered their state of health was either good (45.1%, 276/611) or very good (7.8%, 48/611).

### Use of Information and Communication Technologies When Searching for Health-Related Information

**Figure 1:**
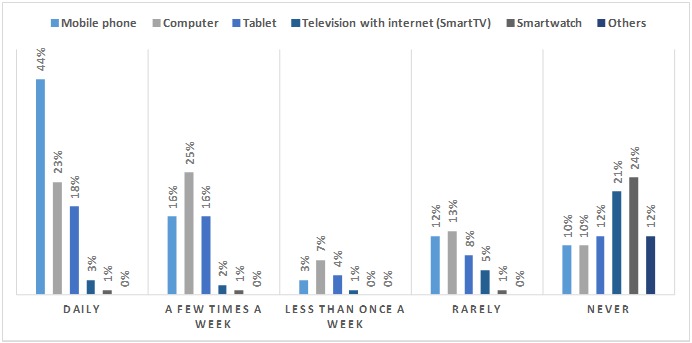
Frequency with which patients used different information and communication technologies to search for information on the internet.

Figure 1 shows the frequency with which patients used different ICTs to search for information on the internet. With regard to health-related information, 87.1% (532/611) of patients were interested in staying informed about health issues. Of all subjects, 75.5% (462/611) sought information from health professionals and 61.3% (375/611) on the internet. Of these, 71.2% (436/611) searched through Google and 21.9% (134/611) by means of social networks (YouTube, Twitter, and Facebook, among others), 20.3% (124/611) used medical societies, and 20.1% (123/611) sought information via patient-advocacy groups.

In response to the question concerning the reasons why patients looked for health information, 49.2% (301/611) replied they wanted to obtain information about disease prevention, healthy lifestyles, and health care, and 47.2% (289/611) were seeking information about the treatment their doctor had prescribed for them. We observed statistically significant differences depending on the level of education, age, sex, and tumor type. First, patients with secondary or university education and younger patients searched for more information on health (*P*<.001). Women sought more information regarding alternative medicines (*P*<.001) and how to get in touch with other people with similar health problems (*P*=.04). Patients with breast cancer were more likely to seek information about their treatment (*P*=.03) and alternative medicines (*P*<.001) than those with other types of tumor.

As for the question regarding whether the information they found on the internet was easy to understand, 10.8% (66/611) of patients replied “always.” A higher proportion of young patients and those with secondary or university education responded “always” than those in other demographic groups (*P*<.001). For the question as to whether participants trusted the information they found on the internet, overall 21.1% (129/611) responded “no,” but this was a statistically more frequent reply among older patients (*P*<.001).

Before going to their doctor’s appointment, 21.8% (133/611) of patients turned to the internet for information about their disease and/or treatment, an action that was more common in younger participants (*P*<.001). After their appointment, 50.9% (311/611) of participants referred to the internet, again this was more typical among younger patients, as well as those with secondary or university education (*P*<.001). The rest of the responses to questions covering the use of ICTs when looking for information on health issues are detailed in Table 2.

### Usage Preferences for Health Apps

Table 3 shows the results for preferences regarding the use of health apps. Of all participants surveyed, 82.7% (505/611) had a smartphone and 20.3% (124/611) had a health app. With respect to mobile phone use, 56.7% (347/611) of participants used theirs to access the internet and 42.8% (262/611) to use apps, among other functions. Of the variables analyzed here, we found that younger patients used apps to obtain information about their illness and its treatment (*P*=.01) and to manage their medical appointments and record and monitor their symptoms (*P*<.01) with a greater frequency than the other sociodemographic groups. Furthermore, patients with secondary or university education were more likely to use apps for disease prevention, health problems, and improving their lifestyle (*P*=.004), as well as for managing their appointments with the health care center or health professional (*P*=.03) than patients with different levels of education.

**Figure 2:**
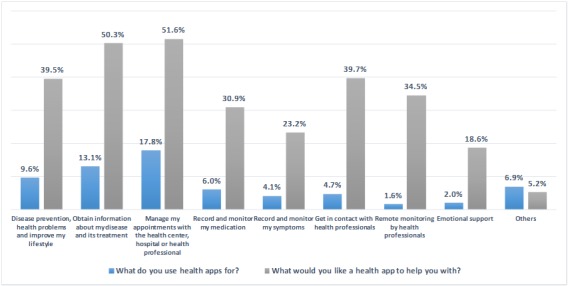
Reasons why patients used a health app, and things they wish the app could help them with.

Figure 2 presents the reasons why our patients used health apps and their usage preferences. We observed that patients with secondary and/or university education were statistically more inclined than other the patient groups to use apps to manage medical appointments (*P*<.01), record and monitor their medication (*P*<.01), get in contact with health professionals (*P*<.01), and facilitate their remote monitoring (*P*=.02).

**Table 1:** Sociodemographic characteristics of patients (N=611).

Demographic characteristics		Patients, n (%)
**Sex**		
	Male	233 (38.1)
	Female	378 (61.9)
**Age in years**		
	<55	256 (41.9)
	56-65	179 (29.3)
	>65	176 (28.8)
**Lives alone?**		
	Yes	61 (10.0)
	No	547 (89.5)
	No response	3 (0.5)
**Makes use of a telehomecare system (social nature service that facilitates daily life activities of dependents using technology)?**		
	No	576 (94.3)
	Yes	35 (5.7)
**Level of education**		
	No education or incomplete primary education	32 (5.2)
	Primary education	110 (18.0)
	Secondary education	220 (35.9)
	University education	249 (40.7)
**Some degree of dependence?**		
	No	528 (86.4)
	Needs help once a day	29 (4.7)
	Needs help several times a day	39 (6.4)
	Totally dependent	14 (2.3)
	No response	1 (0.2)
**Type of cancer**		
	Bladder	9 (1.5)
	Breast	124 (20.3)
	Cervical	8 (1.3)
	Colorectal	113 (18.5)
	Esophageal	13 (2.1)
	Head and neck	16 (2.6)
	Hematological	179 (29.2)
	Kidney	16 (2.6)
	Lung	60 (9.8)
	Ovarian	31 (5.1)
	Pancreatic	28 (4.6)
	Prostate	12 (2.0)
	Sarcoma	16 (2.6)
	Skin or melanoma	8 (1.3)
	Stomach	11 (1.8)
	Testicular	6 (1.0)
**How would you rate your overall health?**		
	Very good	48 (7.9)
	Good	276 (45.2)
	Average	226 (37.0)
	Poor	50 (8.2)
	Very poor	8 (1.3)
	No response	3 (0.2)

**Table 2:** Frequency of using new technologies to consult internet for oncohematological patients (N=611).

Use of information and communication technologies		Patients, n (%)
**Which of the following terms have you heard before? (multiple answers are allowed)**		
	Mobile app	413 (67.5)
	Smartphone	404 (66.0)
	information and communication technologies	179 (29.2)
	I have not heard any of these terms before	142 (23.2)
	eHealth	88 (14.4)
	Mobile health or mHealth	66 (10.8)
	Wearable	51 (8.3)
**Which devices do you use to look for information on the internet? (multiple answers are allowed)**		
	Mobile phone	445 (72.7)
	Desktop and/or laptop computer	416 (68.0)
	Tablet	274 (44.8)
	Television with internet (SmartTV)	58 (9.5)
	Smartwatch	15 (2.5)
	Others	3 (0.5)
	I do not look for information on the internet	84 (13.7)
**Are you interested in staying informed about health-related matters?**		
	Yes	532 (87.1)
	No	60 (9.8)
	No response	19 (3.1)
**Where do you search for information on health? (multiple answers are allowed)**		
	Health professionals	462 (75.5)
	Internet	375 (61.3)
	People close to me (friends, relatives, and workmates)	160 (26.1)
	Newspapers, magazines, and pamphlets	151 (24.7)
	Mobile apps	62 (10.1)
	Others	23 (3.8)
**If you use the internet to search for health information, which types of website do you use? (multiple answers are allowed)**		
	Google	436 (71.2)
	I do not search for medical information on the internet	129 (21.1)
	Medical societies	124 (20.3)
	Patient associations	123 (20.1)
	YouTube	68 (11.1)
	Facebook	45 (7.4)
	Blogs	43 (7.0)
	Others	21 (3.4)
	Other social networks	11 (1.8)
	Twitter	10 (1.6)
**For what purposes do you search for health information? (multiple answers are allowed)**		
	Disease prevention, healthy lifestyles, and health care	301 (49.2)
	To find information about the treatment prescribed by my doctor	289 (47.2)
	To find symptoms and learn about potential diseases	145 (23.7)
	To find information about medical centers or health professionals	139 (22.7)
	To find information about alternative or complementary medicines (herbal products and acupuncture)	107 (17.5)
	To get in contact with other people with health problems such as mine	71 (11.6)
	Others	69 (11.3)
**Is it easy to understand the health information you find on the internet?**		
	Usually	269 (44.0)
	Sometimes	148 (24.2)
	No response	77 (12.6)
	Always	66 (10.8)
	Never	51 (8.3)
**Do you trust the health information you find on the internet?**		
	Yes	84 (13.7)
	No	129 (21.1)
	Depends on the website	336 (55.0)
	No response	62 (10.1)
**Do you look up information on the internet about your disease and/or treatment *before* going to your doctor’s appointment?**		
	No	451 (73.8)
	Yes	133 (21.8)
	No response	27 (4.4)
**Do you look up information on the internet about your disease and/or treatment *after* going to your doctor’s appointment?**		
	Yes	143 (23.4)
	Only if I have still got doubts about something	168 (27.5)
	No	270 (44.2)
	No response	30 (4.9)

**Table 3:** Usage preferences for health apps (N=611).

Usage preferences for health apps		n (%)
**Is your mobile phone a smartphone?**		
	No	93 (15.2)
	Yes	505 (82.7)
	No response	13 (2.1)
**Do you have health-related apps installed?**		
	No	478 (78.2)
	Yes	124 (20.3)
	No response	9 (1.5)
**What do you use your mobile phone for? (multiple answers are allowed)**		
	Normal phone use (calls, messages, and photos or videos)	591 (96.6)
	To access the internet	347 (56.7)
	Schedule planner and alarms	305 (49.8)
	To use apps	262 (42.8)
	Social networks	233 (38.1)
**How would you like to communicate with your health professional? (multiple answers are allowed)**		
	Telephone	470 (76.8)
	Email	270 (44.1)
	Mobile apps	250 (40.8)
	Videoconference	116 (19.0)
	Website	51 (8.3)
	Blogs	20 (3.3)
	Social networks	18 (2.9)
**Would you use an app if your health professional recommended it?**		
	Yes	498 (81.5)
	No	82 (13.4)
	No response	31 (5.1)
**Would you download a health-related app if you had to pay approximately €2.15?** (**considered the average price of the app aimed at patients with cancer)**		
	No	242 (39.6)
	Probably	199 (32.6)
	Yes	102 (16.7)
	No response	68 (11.1)

## Discussion

### Principal Findings and Comparison With Prior Work

To our knowledge, this is the first survey aimed to identify the needs and interests of hematology-oncology patients in the ICT as a means of managing their disease. In this survey conducted in 611 patients, we observed that most of the patients (505/611, 82.7%) owned a smartphone. Furthermore, almost half of patients use their mobile, which was the most popular device among the different options, to perform internet searches every day.

### Use of Information and Communication Technologies When Searching for Health-Related Information

We have seen that health is a cause for concern for 86.9% (531/611) of our patients. Traditionally, patients have tried to physically obtain medical information from ordinary health professionals (doctors, pharmacists, and nurses) [2,4,13]. However, the expansion and generalization of the use of ICTs has implied a radical change in how we obtain information about health and manage diseases [3,4,5,12,14]. Despite the fact that professional health care is still the main resource to obtain information on the pathology, 61.3% (375/611) of our patients turn to the internet to perform a query; similar data were reported by LC et al [15].

In addition, in this study, we have noted that 21.8% (133/611) of patients look up facts about their disease before visiting the doctor, a figure that can rise to 50% after their appointment. These data are akin to those reported by ONTSI [4]. This highlights the growing interest shown by patients and could represent a problem because of the heterogeneous nature of the data. The internet, without the necessary knowledge, is not the most reliable source of health-related information [10,13]. Of all participants in our survey, 71.2% (436/611) used search engines such as Google, and 10.1% (62/611) employed apps to look for information. However, only 10.8% (66/611) always found the information easy to understand. In this regard, the health professional must assume a proactive role when recommending the most reliable websites or apps and adapt them to the understanding of a patient [3]. According to the ONTSI, 38.9% of patients would like their doctor to recommend devices or apps to help them manage their health [4], yet only 10% to 20% of health professionals recommend websites, devices, or apps to their patients [4,16].

### Usage Preferences for Health Apps

If we analyze interests of our patients, more than 40% (244/611) would like to be able to communicate with their health professional by means of an app or via email, a figure close to the 37.3% detailed in the ONTSI report published in April 2016 [4]. At this time, only 20.3% (124/611) of patients have a health-related app although 81.5% (498/611) would use them if recommended by their health care provider. There must be a certain guarantee about the accuracy and quality of the information on the apps, so this does not become a barrier for their own prescription. In a review of apps focused on cancer patients, it was found that only 48.8% were developed with the participation of a health care organization, only half of the apps had been updated in the last year, while 30% had not been updated for over 2 years [3].

Despite the interest these new technologies generate, the cost is a key factor governing their use. Considering the average price of apps designed for cancer patients is €2.15 [3], half of all patients would not download them if they had to pay this amount. Probably, this is because there is a culture among Spanish patients and relatives that everything provided by hospitals is paid for by the social security system and they have never had to pay for anything.

Finally, it is worth highlighting that although the world of apps is evolving considerably, they do not actually cover all our patients’ needs [17,18]. Obtaining information about a disease or its treatment would interest 50% of our patients, which is more than the 28.1% reported by Soler et al [19]; however, less than 20% used apps for this purpose. Moreover, remote monitoring by health professionals is currently one of the least used functions, although it has the greatest potential for use [20-23], as 1 in 3 patients would like to have an app with this feature. Providing a new means of communication through ICTs could offer patients the opportunity to get involved in their disease and assume more responsibility with regard to monitoring their health, thus promoting patient empowerment [5,6,10,24,25]. That is why an easy-to-use app would prove to be a complete tool for this type of patient [3,5,6,13]. To date, existing apps for cancer patients concentrate solely on isolated problems—they do not cover all the needs associated with hematology-oncology patients [3,21-24].

### Limitations

The survey is a type of quantitative research to obtain a large amount of information that objectively can be extrapolated. However, there are other qualitative techniques such as interviews or discussion groups that allow us to know in-depth the perceptions of patients. This type of technique can complete the surveys so that the analysis of the results is much more complete. The results of this survey are being taken into account in a larger project that we are carrying out. The aim of this project was to improve the integral circuit of oncohematological patient care. The semistructured interview methodology with groups of patients is being used. Another limitation of the survey was the number of questions, which could complicate its degree of fulfillment. The questionnaire was therefore handed out at the oncology day hospital so that patients could complete it while they received chemotherapy. Participants could also take the questionnaire home with them and return it completed at their next appointment that resulted in a very low percentage of dropouts. On the other hand, as the survey included new terms that were unfamiliar to some participants, they may not have understood some of the questions while completing the questionnaire. To overcome this limitation, the survey was mainly conducted in the oncology day hospital or outpatient clinic where collaborating researchers were available to help resolve any doubts patients may have had. The questionnaire also included a telephone number patients could call to clear up any doubts or questions that may arise while completing it at home. Finally, questions were completed by patients in Spain in the day hospital or the pharmaceutical care consultancy at pharmacy services. Thus, part of the results may not be representative of other cancer patients in different health systems. However, because we have described exhaustively the working methodology and the complete survey has been published, this work could be reproduced at any center.

### Conclusions

The results of the survey show that hematology-oncology patients are very interested in finding health-related information via ICTs, especially using smartphones and apps. These apps, which are presented as tools that cover these needs, are used as both a source of information and as a new communication channel between patients and health professionals. Free access to apps is still an important factor when it comes to promoting their use and reaching a greater number of users. The app features that draw the most interest are appointment management, advice on disease management, and personalized communication with health professionals. However, many of the apps available have not been validated or updated and do not offer any alternatives to medical care. Therefore, the health professional plays an essential role in recommending and validating these tools to ensure they are of high quality standards.

## Abbreviations

ICT: information and communication technology
ONTSI: Spanish National Observatory of Telecommunications and Information Society
